# Epidemiology of Human Cryptosporidiosis in Brazil: A Systematic Review Highlighting *Cryptosporidium parvum*

**DOI:** 10.3390/tropicalmed10110313

**Published:** 2025-10-31

**Authors:** João Victor Inácio Santos, Welitânia Inácia Silva, Basílio Felizardo Lima Neto, Thais Ferreira Feitosa, Vinícius Longo Ribeiro Vilela

**Affiliations:** 1 Postgraduate Program in Science and Animal Health, Federal University of Campina Grande—UFCG, Patos 58055-018, PB, Brazil; jjvsantos987@gmail.com (J.V.I.S.); welitaniais20@gmail.com (W.I.S.); felizardobasilio95@gmail.com (B.F.L.N.); thais.feitosa@ifpb.edu.br (T.F.F.); 2 Department of Veterinary Medicine, Federal Institute of Paraíba—IFPB, Sousa 58805-345, PB, Brazil

**Keywords:** *Cryptosporidium* spp., neglected diseases, molecular epidemiology, vulnerable populations, zoonoses

## Abstract

Cryptosporidiosis is a zoonotic disease of medical and veterinary importance caused by *Cryptosporidium* spp. This study conducted a systematic review to assess the occurrence and distribution of *Cryptosporidium* spp. in humans in Brazil, with emphasis on *C. parvum*. Following the PRISMA (Preferred Reporting Items for Systematic Reviews and Meta-Analyses) protocol and using five databases, 3689 articles were screened, and 48 met the inclusion criteria. Most studies were concentrated in the Southeast Region, particularly São Paulo, while major gaps were identified in the North and Midwest Regions. The mean prevalence was 8.9% using direct methods and 52.2% using indirect methods, with the highest positivity reported in the Northeast Region. Microscopy was the most frequently employed diagnostic tool, although it showed limited ability to differentiate species. When combined with molecular approaches, *C. parvum* and *C. hominis* were identified as the predominant species. Infection was most common among children and immunocompromised individuals, especially those with HIV and kidney diseases. Overall, the findings highlight substantial research gaps regarding cryptosporidiosis in Brazil and its disproportionate impact on vulnerable populations. Expanding regional studies, integrating molecular methods for species characterization, and implementing targeted public health strategies are essential to improve epidemiological knowledge and guide prevention and control measures.

## 1. Introduction

*Cryptosporidium* spp. is a zoonotic protozoan belonging to the phylum Apicomplexa, widely recognized for its pathogenic potential and as one of the leading causes of diarrhea, particularly in immunosuppressed individuals [1]. This parasite is commonly found in the gastrointestinal tract of various hosts and stands out as one of the most prevalent parasites transmitted on a global scale [2]. The *Cryptosporidium* genus currently comprises at least 44 described species, widely recognized for their important role in the etiology of enteric infections in humans and a wide range of animals. These parasites are responsible for diarrheal diseases of global significance in both public and veterinary health [3,4,5]. Among them, *Cryptosporidium hominis* and *Cryptosporidium parvum* are the main species associated with cryptosporidiosis in humans [6,7].

Considered an opportunistic pathogen of medical and veterinary relevance, *Cryptosporidium* spp. plays an important role in the development of enteric diseases in vulnerable groups, such as children, the elderly, and immunocompromised individuals, including patients living with HIV, undergoing cancer treatment, or receiving transplants [8]. In these groups, infection may present with severe clinical manifestations and, in some cases, be fatal. In patients with AIDS, cancer, or on hemodialysis, for example, acute diarrhea is common and often associated with high morbidity and mortality [9]. In contrast, in immunocompetent individuals, infection tends to be self-limiting, with spontaneous resolution and lower clinical severity [10,11,12].

Transmission occurs through the shedding of viable oocysts in the feces of infected hosts. These oocysts, which contain four infectious sporozoites, are immediately capable of initiating a new infection without requiring maturation in the environment [1]. This characteristic facilitates dissemination through direct contact with infected people or animals, as well as through indirect routes such as contaminated water, food, and fomites [13]. The thick oocyst wall provides prolonged resistance under different environmental conditions, which significantly increases the risk of exposure and favors outbreaks through both human-to-human and zoonotic transmission [14,15].

Most cases of human cryptosporidiosis are attributed to *C. parvum*, recognized for its zoonotic potential. To a lesser extent, *C. hominis*, which is adapted to the human host, is also relevant. Together, these two species account for more than 90% of reported human cases and are frequently associated with epidemic outbreaks in several countries. Moreover, other species such as *Cryptosporidium meleagridis*, *Cryptosporidium felis*, and *Cryptosporidium canis*, although less frequent, have been detected in human cases, indicating the occurrence of zoonotic transmission and the diversity of infection sources [4,16,17,18].

Although the impacts of human cryptosporidiosis are well documented internationally, significant gaps remain in Brazil regarding knowledge of the infection’s epidemiology, particularly with respect to geographic distribution, the most affected population groups, the diagnostic methods used, and the molecular characterization of species and subtypes involved. Considering Brazil’s socio-environmental diversity, inequalities in access to basic sanitation, and the importance of surveillance of emerging diseases with zoonotic potential, it is essential to compile and critically analyze the available data. Due to its ability to affect the health of both humans and animals, causing significant impacts on public health and economic losses in animal production, *C. parvum* stands out as particularly relevant in the Brazilian context. Therefore, this systematic review aims to gather evidence contributing to the understanding of the occurrence and distribution of *C. parvum* in humans in Brazil, providing support for more effective surveillance, prevention, and control actions.

## 2. Materials and Methods

This systematic review followed the standard PRISMA 5 protocol (Preferred Reporting Items for Systematic Reviews and Meta-Analyses) for the design, reporting, and interpretation of the results [19]. The protocol was previously registered in the international PROSPERO database under the number CRD420251131994.

### 2.1. Search Strategies

The central research question of this systematic review was structured using the PEO framework: Population—humans in Brazil; Exposure—infection by *Cryptosporidium parvum*; Outcome—occurrence of infection, genotypes and subtypes, clinical impacts, comorbidities, risk factors, zoonotic potential, and diagnostic methods.

For the present study, a comprehensive search was conducted across five distinct databases: Web of Science, Google Scholar, ScienceDirect, Scopus, and PubMed. All retrieved articles were related to *C. parvum* infections in humans, published in Brazil up to May 2025, with no restrictions regarding geographic location or language. The search was performed on 21 May 2025, using keywords, indexed terms, or title words, based on the following combination: (*Cryptosporidium* spp. OR *Cryptosporidium parvum* OR Cryptosporidiosis) AND (Human) AND (Brazil).

### 2.2. Inclusion Criteria and Selection Process

After retrieval, articles from the databases were imported into BibTeX files using Mendeley Reference Manager version 2.112.0, and duplicates were removed. The initial selection was based on title and abstract screening.

Inclusion criteria comprised articles and case reports reporting studies on the prevalence, frequency, and occurrence of *C. parvum* in humans in Brazil, containing both the total sample size and the exact number of positive cases. The reference lists of selected articles were also examined to identify additional studies that might not have been detected in the initial search.

Exclusion criteria included review articles, conference proceedings, studies without participation of *Cryptosporidium* spp., studies conducted exclusively in animals, studies without access to full text, experimental works evaluating only diagnostic methods, studies carried out outside Brazil, and academic theses or dissertations.

Two reviewers independently screened the articles. In cases of disagreement regarding the inclusion of a study, a third reviewer was consulted to decide on its inclusion or exclusion, whose decision was considered final.

### 2.3. Data Extraction and Analysis

All eligible articles were downloaded in full, and their data were recorded in Microsoft Excel^®^, version 2016. Articles were organized into spreadsheets containing the following epidemiological information: author, age, sex, total number of samples, number of positive samples, prevalence, diagnostic method, species, sequencing and genotyping, state, region, study type, presence of comorbidities in the studied population, type of comorbidity, and risk factors. Subsequently, a descriptive analysis of the extracted data was performed, without meta-analysis or statistical testing, due to the heterogeneity among the studies. Comparisons between variables were presented in an exploratory manner, without formal statistical inference.

## 3. Results

**Figure 1:**
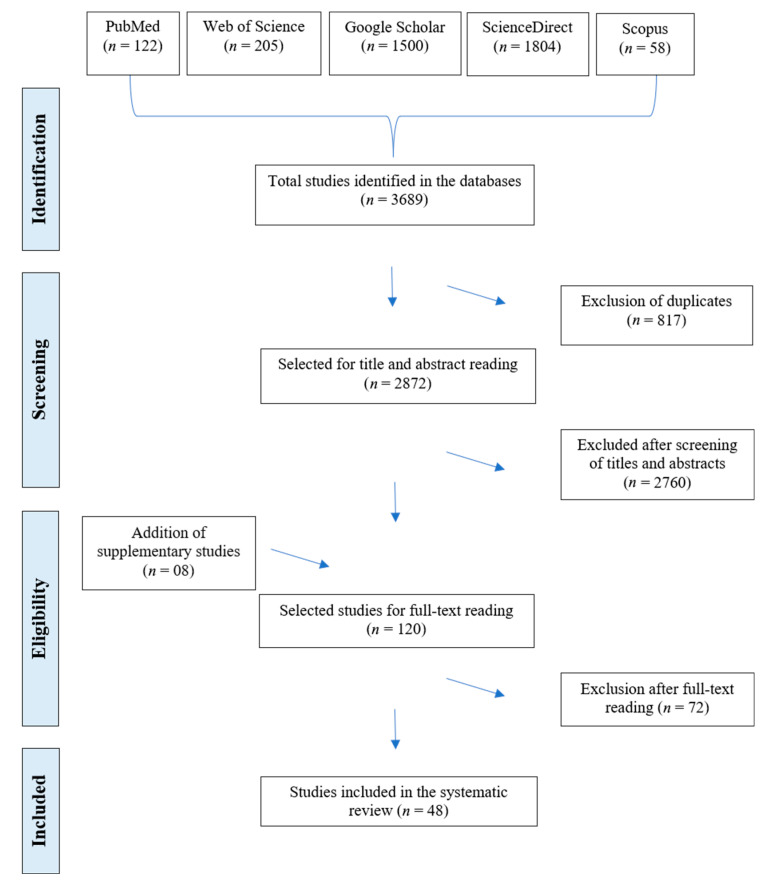
PRISMA (Preferred Reporting Items for Systematic Reviews and Meta-Analyses) flowchart, inclusion and exclusion criteria for articles on *Cryptosporidium* spp. infection in humans in Brazil.

Initially, 3689 studies were identified in the databases consulted. After removing duplicates (*n* = 817), screening titles and abstracts (*n* = 2760), and including complementary studies (*n* = 8), 120 articles were selected for full-text evaluation. Of these, 72 were excluded after full-text screening for not meeting the predefined methodological criteria. In total, 48 studies were included in the present systematic review (Figure 1).

**Figure 2:**
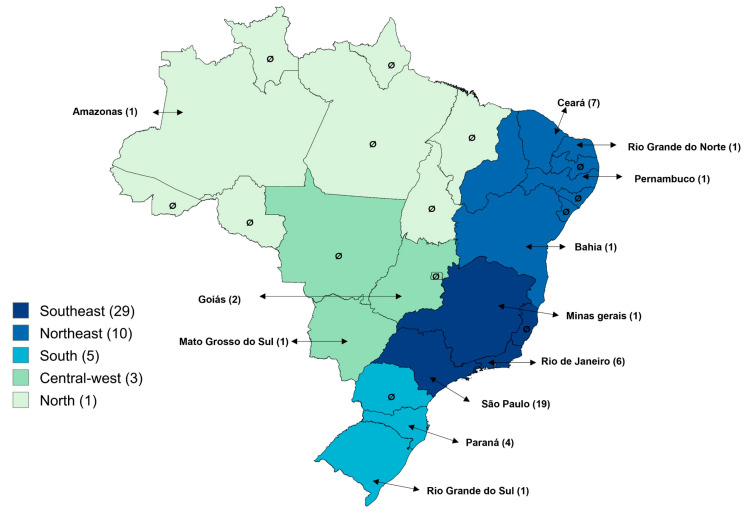
Distribution of articles on *Cryptosporidium* spp. infections in humans across regions and states of Brazil.

The geographic distribution of studies on *Cryptosporidium* spp. infections in humans across different regions and states of Brazil is presented in Figure 2. A predominance of publications was observed in the Southeast Region (60.4%; 29/48), followed by the Northeast (20.8%; 10/48), South (10.4%; 5/48), Midwest (6.3%; 3/48), and North (2.1%; 1/48). Among the states, São Paulo stood out with 39.6% (19/48) of the studies, followed by Ceará (14.6%; 7/48), Rio de Janeiro (12.5%; 6/48), Paraná (8.3%; 4/48), and Minas Gerais (8.3%; 4/48). The states of Amazonas, Mato Grosso do Sul, Goiás, Bahia, Pernambuco, Rio Grande do Norte, and Rio Grande do Sul each had only one study (2.1%; 1/48). No publications were identified in the remaining 14 states or in the Federal District.

The main information from the prevalence studies, including study location, age group, observed prevalence, diagnostic methods employed, identified genus or species, and study design, is presented in Table 1.

Table 2 presents the distribution of *Cryptosporidium* spp. prevalence across different regions of Brazil, considering the total number of samples analyzed and the positive cases, case reports were excluded from the analysis. In total, 11.761 samples were examined using direct diagnostic methods, of which 1.042 (9%) tested positive, and 1.158 samples were analyzed using indirect methods, with 605 (52.2%) testing positive. It is important to note that, in some studies, the same sample was tested using more than one diagnostic technique, which explains variations in the total number of samples and positive results. The Southeast region accounted for the largest number of samples analyzed by direct (7.943; 65.6%) and indirect (856; 73.9%) methods, whereas the highest prevalence was recorded in the Northeast, with 18% positivity by direct methods and 83.8% by indirect methods.

Table 3 presents the main information from case reports of human infections by *Cryptosporidium* spp. in Brazil, including study location, age group, observed prevalence, diagnostic methods employed, identified genus or species, and study design.

For the diagnosis of *Cryptosporidium* spp., the studies employed direct and indirect methods, sometimes in combination. Among the direct methods, microscopy was the most frequently used, reported in 91.6% (44/48) of articles, with 60.4% (29/48) using it alone. The combination of microscopic and molecular diagnosis was applied in 18.8% (9/48) of studies. Histopathology was used in 4.1% (2/48) of the studies, direct immunofluorescence combined with microscopy in 2.1% (1/48), and immunochromatography in 2.1% (1/48) of the articles. The only indirect method employed was enzyme-linked immunosorbent assay (ELISA), used alone in 2.1% (1/48) of studies or in combination with microscopy in 10.4% (5/48) of studies. The prevalence obtained by the different diagnostic methods, used alone or in combination, is presented in Table 4, comprising a total of 53 datasets.

**Figure 3:**
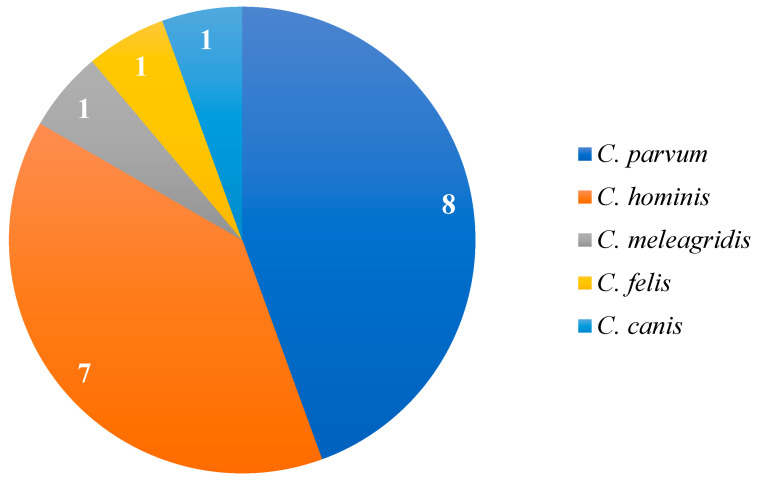
Frequency of *Cryptosporidium* species reported in studies that employed molecular diagnosis based on the 18S rRNA and gp60 genes for species identification.

Molecular analyses enabled specific detection, primarily of *C. parvum* (88.9%; 8/9) and *Cryptosporidium hominis* (77.8%; 7/9). The frequency of species identified in the studies is shown in Figure 3. Genotyping was performed in only one study, Cunha et al. [12], which identified the subtype IIaA15G2R1.

The analysis demonstrated a predominance of cross-sectional studies, which accounted for 66.7% (32/48) of the articles. Longitudinal studies represented 16.7% (8/48), mainly used for clinical follow-up of vulnerable populations and temporal outbreak surveillance. Case reports also accounted for 16.7% (8/48) of the total analyzed articles.

Information on participants’ age was reported in 81.2% (39/48) of the studies. Among these, 41% (16/39) focused exclusively on infections in children, corresponding to 52.6% (555/1056) of positive samples by direct methods and 3.5% (21/605) by indirect methods.

Regarding health conditions, 63.5% (30/48) of the studies included individuals with comorbidities. Most investigations focused on populations with immunosuppression or chronic diseases, representing 93.3% (28/30) of these studies. This group included people living with HIV, cancer patients, recipients of kidney or bone marrow transplants, patients on renal replacement therapy, individuals with chronic kidney disease, and patients admitted to intensive care units (ICUs). Together, these studies accounted for 38% (401/1056) of positive samples by direct methods and 7.4% (45/605) by indirect methods.

The main risk factors identified were related to clinical and immunological conditions, reported in 39.5% (19/48) of studies that investigated determinants associated with *Cryptosporidium* spp. infection. These factors accounted for 54% (572/1056) of positive samples detected by direct methods and 15% (91/605) by indirect methods. The most prevalent factor reported was diarrhea, described in 47.3% (9/19) of articles, responsible alone for 56.2% (322/572) of positive samples, all identified by direct methods. Diarrhea was consistently associated with greater clinical severity, weight loss, low body mass index, and delayed child development, highlighting its central role as a marker of the most critical clinical manifestations of infection.

## 4. Discussion

This systematic review compiled and analyzed relevant data on the occurrence of *Cryptosporidium* in humans in Brazil, providing a more comprehensive view of the national epidemiological situation. The mean prevalence obtained in this review (9%) is consistent with prevalences reported in other South American countries, such as 7% in Peru and 13% in Argentina and Venezuela [67]. These variations may reflect methodological, population, and environmental differences, but overall, they demonstrate the widespread distribution of *Cryptosporidium* in South America, highlighting its extensive presence across the American continent.

In Brazil, most studies were conducted in the Southeast Region, particularly in the state of São Paulo, areas with higher economic capacity and scientific infrastructure, and consequently greater research support [68]. However, the limited number of studies in the Midwest and North regions reveals the uneven distribution of epidemiological information on *Cryptosporidium* spp. in humans in the country. This data disparity hinders the accurate interpretation of the pathogen’s true distribution in Brazil and the development of adequate public health policies and surveillance strategies to address vulnerable populations across all regions. In addition, the higher concentration of economic resources in regions such as the Southeast, compared to lower-income areas like the Midwest and North, reflects disparities in access to healthcare services and, consequently, in disease diagnosis, resulting in underreporting of cases in specific regions.

Microscopy was the most commonly used method in the majority of studies; however, it is important to highlight that its low sensitivity may compromise the accuracy of the results. Although it is a practical and relatively simple method, microscopy has a crucial limitation: it only allows identification of the parasite at the genus level and cannot differentiate species or subtypes of *Cryptosporidium* spp. [46]. Due to this limitation, morphologically similar species such as *C. parvum* and *C. hominis* may be misclassified when microscopy is used as the sole diagnostic method, hindering the accurate identification of the etiological agent involved in each infection. This represents a critical factor affecting the reliability of the data and the proper understanding of the epidemiology of cryptosporidiosis. This limitation is critical for the reliability of the data and for a proper understanding of the infection’s epidemiology. Nevertheless, six studies diagnosed *C. parvum* using microscopy alone [22,23,24,39,49,50,63]. Most of these studies were published some time ago, when molecular and immunological methods were not widely available, which may have limited the accuracy of species identification.

Combining microscopy with higher-accuracy methods, such as molecular techniques, increases diagnostic precision and sensitivity for different *Cryptosporidium* spp. species and subtypes [12]. The data showed that although *C. parvum* and *C. hominis* are the most common species in human infections, other species, such as *C. meleagridis*, *C. felis*, and *C. canis*, can also infect humans. Some species may be more pathogenic than others, emphasizing the importance of methods capable of accurately distinguishing the etiological agent to guide appropriate treatment [47].

Few studies employed genetic sequencing and genotyping methods. This limitation is partly due to the absence of such assays in routine laboratory practice, combined with high costs, the need for specialized infrastructure, and the demand for trained personnel [69]. In Brazil, only one study molecularly characterized *C. parvum* [12], identifying the IIaA15G2R1 subtype in humans, which is frequently associated with infections in cattle in the country [5], reinforcing its zoonotic transmission potential. This subtype is also among the most prevalent in European studies, being identified in both human outbreaks and livestock in the United Kingdom [70]. Subtype identification is essential to deepen understanding of the parasite’s behavior in the host, providing valuable information for developing more effective strategies for prevention and treatment of cryptosporidiosis [71]. This knowledge gap hampers the early identification of more virulent or epidemiologically significant subtypes, delaying the implementation of effective and context-specific control measures.

Other direct and indirect methods, such as ELISA, were also reported. Although serological methods have good sensitivity and specificity for the diagnosis of *Cryptosporidium* spp., molecular methods offer greater advantages for prevalence studies and diagnostic confirmation, as they demonstrate 100% sensitivity and specificity, allowing visualization of the individual’s infection status at the time of sampling [12,42,72]. Nevertheless, indirect methods can still be used for epidemiological monitoring of the disease. Therefore, combining different diagnostic methods allows the identification of the parasite at different stages of infection.

In studies reporting age groups, 41% focused exclusively on children, possibly due to the higher susceptibility of this group to *Cryptosporidium* spp. infections, as the immune system is not fully developed, and the decline in maternal antibody protection, combined with increased exposure to pathogens through feeding practices, makes this group more vulnerable [54,73]. *C. parvum* infections in children are associated with higher oocyst shedding compared to *C. hominis* infections and are linked to deficits in child growth [54]. This demonstrates that *Cryptosporidium* spp. infections can cause serious health problems in children, including developmental delays.

The results indicate that clinical and immunological factors were the main risk factors investigated in 39.5% of the studies. Among 1056 positive cases detected by direct methods, 54% (572/1056) were associated with these conditions. Chronic diseases that cause immunosuppression, particularly HIV, markedly increase susceptibility to *Cryptosporidium* spp. infections [44,60,74] and lead to more severe clinical outcomes [44] due to the depletion of CD4+ T lymphocytes, essential for controlling intracellular parasites [74]. Therefore, immunosuppression, especially in HIV/AIDS patients, remains a key determinant of disease severity in cryptosporidiosis.

Cryptosporidiosis is a parasitic infection that causes a variety of symptoms, with watery diarrhea, abdominal pain, and weight loss being the most common, and more severe in immunosuppressed individuals [41,53,65]. As shown in the results, diarrhea was the most prevalent factor, present in almost half (47.3%) of the investigated studies and responsible for 19.3% of all positive samples. Diarrhea results from alterations in the intestinal epithelium, such as microvilli destruction, leading to malabsorption and increased fluid secretion, consequently resulting in watery diarrhea [39].

Moreover, the results confirmed that diarrhea is frequently associated with greater clinical severity, weight loss, low body mass index (BMI), and delayed child development. Weight loss, in particular, is a recurrent symptom and is linked to nutrient malabsorption, increased intestinal permeability, and the chronic inflammatory response triggered by the infection [41,53]. These symptoms can have serious health consequences, leading to high treatment costs and posing risks to the patient’s life. It is therefore important to associate infections with the described symptomatology to support early diagnosis and appropriate treatment.

This study has some limitations that should be acknowledged. A potential publication bias that should be highlighted is that most of the included studies were conducted in urban areas and hospital-based settings, while rural and remote regions remain underrepresented. This imbalance may lead to an underestimation of the true burden of *Cryptosporidium* infection in vulnerable populations with limited access to healthcare and diagnostic services. Additionally, the predominant use of microscopy in the included studies represents a methodological limitation, as this technique has lower sensitivity and does not allow species or subtype differentiation. These factors may affect the accuracy of prevalence estimates and the understanding of the parasite’s epidemiological diversity in Brazil.

## 5. Conclusions

This systematic review confirms that cryptosporidiosis is a notable public health concern in Brazil. Most studies were conducted in the Southeast, particularly in São Paulo, with notable gaps in the North and Midwest regions, limiting the development of equitable health policies nationwide. Although microscopy is the most frequently used diagnostic method, it has low sensitivity and limited species differentiation. Molecular methods, though less employed, are crucial for detecting infections by *C. parvum*, *C. hominis*, and less common species such as *C. meleagridis*, *C. felis*, and *C. canis*. The high incidence in children and immunocompromised individuals underscores the urgent need for targeted prevention and control measures. Expanding regional studies, incorporating molecular diagnostics, and implementing public health strategies are essential to protect vulnerable populations from cryptosporidiosis.

## Figures and Tables

**Table 1:** Prevalence studies of *Cryptosporidium* spp. infections in humans in Brazil.

Region and State	Reference	Age	Diagnostic Method	Total of Samples	Positives	Prevalence (%)	Gender or Species	Study Type
Southeast								
São Paulo	Dias et al. [20]	NA	Microscopy	157	19	12.1	*Cryptosporidium* spp.	Crosssectional
São Paulo	Garlipp et al. [21]	2 months to 76 years	Microscopy	111	20	18.0	*Cryptosporidium* spp.	Crosssectional
São Paulo	Franco et al. [22]	2 months to 5 years	Microscopy	930	20	6.4	*C. parvum*	Crosssectional
São Paulo	Chieffi et al. [23]	NA	Microscopy	82	20	24.4	*C. parvum*	Longitudinal
São Paulo	Cimerman et al. [24]	>18 years	Microscopy	200	14	7	*C. parvum*	Crosssectional
São Paulo	Mederios et al. [25]	1 day to 10 years	Microscopy	1836	34	1.8	*Cryptosporidium* spp.	Crosssectional
Rio de Janeiro	Silva et al. [26]	18 to 59 years	Microscopy	90	3	3.3	*Cryptosporidium* spp.	Crosssectional
			Direct ELISA		7	7.7		
São Paulo	Cox and Azevedo, [27]	1 day to 39 years	Indirect ELISA	512	307	60	anti-*C. parvum* antibodies	Crosssectional
São Paulo	Marques et al. [28]	NA	Microscopy	144	8	5.5	*C. parvum*	Crosssectional
			Direct ELISA		9	6.2		
São Paulo	Almeida et al. [29]	1 day to 6 years–21 to 50 years	Microscopy	87	14	16.1	*Cryptosporidium* spp.	Crosssectional
São Paulo	Carvalho et al. [30]	1 to 6 years	Microscopy	279	42	15.5	*Cryptosporidium* spp.	Crosssectional
São Paulo	Mascarini and Donalisio [31]	1 day to 7 years	Microscopy	776	74	9.5	*Cryptosporidium* spp.	Crosssectional
Rio de Janeiro	Costa et al. [32]	6 months to 5 years	Microscopy	193	18	9.3	*Cryptosporidium* spp.	Longitudinal
São Paulo	Rossit et al. [33]	1 to 13 years	Immunochromatography	100	62	62	*C. parvum*	Crosssectional
Minas Gerais	Silva et al. [34]	2 to 72 years	Microscopy	359	31	8.6	*Cryptosporidium* spp.	Crosssectional
São Paulo	Araújo et al. [35]	NA	Microscopy/Molecular (nested PCR)	14	14	100	*C. parvum*, *C. hominis*, *C. meleagridis*	Crosssectional
São Paulo	Lucca et al. [36]	NA	Microscopy/Molecular (nested PCR)	27	27	100	*C. parvum*, *C. hominis*, *C. felis*, *C. canis*	Crosssectional
São Paulo	Cardoso et al. [37]	42 years	Microscopy	500	1	0.3	*C. parvum*	Crosssectional
Rio de Janeiro	Rolando et al. [38]	NA	Microscopy/Molecular (nested PCR)	1207	42	3.5	*C. parvum*, *C. hominis*	Crosssectional
Minas Gerais	Assis et al. [39]	14 to 56 years	Microscopy	120	6	10.1	*Cryptosporidium* spp.	Crosssectional
Minas Gerais	Gil et al. [40]	NA	Microscopy	110	0	0	*Cryptosporidium* spp.	Crosssectional
			Direct ELISA		29	26.3		
Minas Gerais	Girotto et al. [41]	60 to 106 years	Microscopy	293	3	1	*Cryptosporidium* spp.	Crosssectional
São Paulo	Fregonesi et al. [42]	3 to 12 years	Microscopy	17	4	23.5	*Cryptosporidium* spp.	Crosssectional
Rio de Janeiro	Peralta et al. [43]	1 to 75 years	Microscopy/Molecular (nested PCR)	89	20	22.5	*C. parvum*, *C. hominis*	Crosssectional
Rio de Janeiro	Cunha et al. [12]	21 to 75 years	Microscopy/Molecular (nested PCR)	125	12	9.6	*C. parvum*, *C. hominis*	Crosssectional
Rio de Janeiro	Adami et al. [44]	NA	Microscopy/Molecular (nested PCR)	97	6	6.2	*Cryptosporidium* spp./*C. hominis*	Crosssectional
Northeast								
Ceará	Newman et al. [45]	3 months to 81 years	Microscopy	202	48	23.7	*C. parvum*/anti-*C. parvum* antibodies	Longitudinal
			Indirect ELISA		191	94.5		
Ceará	Wuhib et al. [46]	3 months to 68 years	Microscopy	295	24	14.5	*C. parvum*	Crosssectional
Ceará	Agnew et al. [47]	3 to 27 months	Microscopy	59	43	72.8	*C. parvum*	Longitudinal
Ceará	Newman et al. [48]	1 day to 4 years	Microscopy	189	58	31.2	*Cryptosporidium* spp.	Longitudinal
Ceará	Lima et al. [49]	1 day to 3 years	Microscopy	762	74	9.8	*Cryptosporidium* spp.	Longitudinal
Ceará	Brantley et al. [50]	21 to 46 years	Microscopy/Molecular (nested PCR)	12	5	41.7	*C. parvum*	Crosssectional
Ceará	Bushen et al. [51]	1 day to 5 years	Microscopy/Molecular (nested PCR)	157	24	26.7	*C. parvum*, *C. hominis*	Longitudinal
Bahia	Teixeira et al. [52]	1 day to 35 years	Microscopy	332	0	0	*Cryptosporidium* spp./anti-*Cryptosporidium* spp. antibodies	Crosssectional
			Indirect ELISA		62	62		
Pernambuco	Nascimento et al. [53]	1 to 14 years	Microscopy	182	59	32.4	*Cryptosporidium* spp.	Crosssectional
South								
Rio Grande do Sul	Jeske et al. [54]	20 to 85 years	Microscopy	73	9	13.3	*Cryptosporidium* spp.	Crosssectional
Midwest								
Mato Grosso do Sul	Oshiro et al. [55]	1 day to 59 months	Microscopy	1051	12	1.1	*C. parvum*	Crosssectional
Goiás	Pereira [56]	21 days to 10 years	Microscopy	445	64	14.4	*C. parvum*	Crosssectional
			DIF		83	18.7		
Goiás	Barcelos et al. [57]	21 to 41 years	Microscopy	90	1	1.1	*Cryptosporidium* spp.	Crosssectional
North								
Amazonas	Loureiro et al. [58]	1 to 2 years	Microscopy	201	6	5.2	*Cryptosporidium* spp.	Longitudinal

Abbreviations: NA, not available; ELISA, Enzyme-Linked Immunosorbent Assay; DIF, Direct Immunofluorescence.

**Table 2:** Distribution of *Cryptosporidium* spp. prevalence across different regions of Brazil, according to the total number of samples analyzed and positive cases. Case reports were excluded from the analysis.

Regions	Total of Samples	Positive Samples	Prevalence (%)
Direct Methods			
Southeast	7943	514	6.5
Northeast	1958	353	18
Midwest	1586	160	10.1
South	73	9	12.3
North	201	6	3
Total	11,761	1042	9
Indirect Methods			
Southeast	856	352	41.1
Northeast	302	253	83.8
Total	1158	605	52.2

**Table 3:** Case reports of human infections by *Cryptosporidium* spp. in Brazil.

Region and State	Reference	Age	Diagnostic Method	Total of Samples	Positives	Prevalence (%)	Gender or Species
Southeast							
São Paulo	Clemente et al. [59]	64 years	Histopathology	1	1	100	*Cryptosporidium* spp.
São Paulo	Souza et al. [60]	43 years	Histopathology	1	1	100	*Cryptosporidium* spp.
São Paulo	Reina et al. [61]	44 to 59 years	Microscopy/Molecular (nested PCR)	2	2	100	*C. parvum*
Northeast							
Rio Grande do Norte	Rezende et al. [62]	60 to 87 years	Microscopy	2	2	100	*Cryptosporidium* spp.
South							
Paraná	Barros et al. [63]	2 to 71 years	Microscopy	20	1	5	*Cryptosporidium* spp.
Paraná	Chiuchetta [64]	33 years	Microscopy	1	1	100	*Cryptosporidium* spp.
Paraná	Cabral et al. [65]	NA	Microscopy	5	5	100	*Cryptosporidium* spp.
Paraná	Rosende et al. [66]	12 years	Microscopy	1	1	100	*Cryptosporidium* spp.

Abbreviations: NA, not available.

**Table 4:** Distribution of diagnostic methods used for the detection of *Cryptosporidium* spp. infections in humans in Brazil.

Direct Methods	Datasets	Total of Samples	Positive Samples	Prevalence (%)
Microscopy	29	8871	616	6.9
Microscopy + Molecular	9	1730	170	9.8
Microscopy + Indirect ELISA *	5	646	59	9.1
Histopathology	2	2	2	100
Microscopy + DIF	1	445	147	33
Immunochromatography	1	100	62	62
Total	47	11,794	1056	9.0
Indirect Methods				
Direct ELISA	1	512	307	60
Microscopy + Indirect ELISA **	5	646	298	46.1
Total	6	1158	605	52.2

Abbreviations: ELISA, Enzyme-Linked Immunosorbent Assay; IFD, Direct Immunofluorescence. * In the results row, only values obtained by microscopy are considered. ** In the results row, only values obtained by indirect ELISA are considered.

## Data Availability

No new data were created or analyzed in this study. Data sharing is not applicable to this article.

## References

[1] Gerace E., Lo Presti V.D.M., Biondo C. (2019). *Cryptosporidium* infection: Epidemiology, pathogenesis, and differential diagnosis. Eur. J. Microbiol. Immunol..

[2] Cunha F.S., Peralta R.H.S., Peralta J.M. (2019). New insights into the detection and molecular characterization of *Cryptosporidium* with emphasis in Brazilian studies: A review. Rev. Inst. Med. Trop. São Paulo.

[3] Zahedi A., Paparini A., Jian F., Robertson I., Ryan U. (2018). *Cryptosporidium* species and subtypes in animals inhabiting drinking water catchments in three states across Australia. Water Res..

[4] Ryan U.M., Feng Y., Fayer R., Xiao L. (2021). Taxonomy and molecular epidemiology of *Cryptosporidium* and *Giardia*—A 50-year perspective (1971–2021). Int. J. Parasitol..

[5] Vilela V.L.R., Feitosa T.F., Silva W.I., Katzer F. (2025). *Cryptosporidium* spp. in livestock in Brazil: An underestimated threat to animal and human health. Curr. Res. Parasitol. Vector-Borne Dis..

[6] Xiao L. (2010). Molecular epidemiology of cryptosporidiosis: An update. Exp. Parasitol..

[7] Khan A., Shaik J.S., Grigg M.E. (2018). Genomics and molecular epidemiology of *Cryptosporidium* species. Acta Trop..

[8] Sannella A.R., Suputtamongkol Y., Wongsawat E., Prasertbun R., Cacciò S.M. (2019). A retrospective molecular study of *Cryptosporidium* species and genotypes in HIV-infected patients in Thailand. Parasites Vectors.

[9] Ahmadpour E., Safarpour H., Xiao L., Zarean M., Hatam-Nahavandi K., Barac A., Picot S., Rahimi M.T., Rubino S., Mahami-Oskouei M. (2020). Cryptosporidiosis in HIV-positive patients and related risk factors: A systematic review and meta-analysis. Parasite.

[10] Danziger-Isakov L. (2014). Gastrointestinal infections after transplantation. Curr. Opin. Gastroenterol..

[11] Siddiqui Z.A. (2017). An overview of parasitic infections of the gastro-intestinal tract in developed countries affecting immunocompromised individuals. J. Parasit. Dis..

[12] Cunha F.S., Jann H.W., Lugon J.R., Peralta J.M., Peralta R.H.S. (2022). Molecular characterization of *Cryptosporidium* spp. obtained from fecal samples of immunosuppressed patients from Brazil. Rev. Soc. Bras. Med. Trop..

[13] Dixon B.R., Parrington L.J., Cook A., Pintar K.D.M., Pollari F., Kelton D., Farber J.M. (2011). The potential for zoonotic transmission of *Giardia duodenalis* and *Cryptosporidium* spp. from beef and dairy cattle in Ontario, Canada. Vet. Parasitol..

[14] Cacciò S.M., Chalmers R.M. (2016). Human cryptosporidiosis in Europe. Clin. Microbiol. Infect..

[15] Smith R.P., Newton K., Rimdap E., Wight A., Robinson G., Chalmers R.M. (2021). A review of investigations of animal premises, linked to human outbreaks of cryptosporidiosis in England and Wales, from 2009–2019. Vet. Rec..

[16] Hadfield S.J., Robinson G., Elwin K., Chalmers R.M. (2011). Detection and differentiation of *Cryptosporidium* spp. in human clinical samples by real-time PCR. J. Clin. Microbiol..

[17] Chalmers R.M., Giles M. (2010). Zoonotic cryptosporidiosis in the UK—Challenges for control. J. Appl. Microbiol..

[18] Delling C., Daugschies A. (2022). Literature review: Coinfection in young ruminant livestock—*Cryptosporidium* spp. and its companions. Pathogens.

[19] Page M.J., McKenzie J.E., Bossuyt P.M., Boutron I., Hoffmann T.C., Mulrow C.D., Shamseer L., Tetzlaff J.M., Moher D. (2021). Updating guidance for reporting systematic reviews: Development of the PRISMA 2020 statement. J. Clin. Epidemiol..

[20] Dias R.M.D.S., Mangini A.C.S., Torres D.M.A.G.V., Corrêa M.O.A., Lupetti N., Corrêa F.M.A., Chieffi P.P. (1988). Cryptosporidiosis among patients with acquired immunodeficiency syndrome (AIDS) in the county of São Paulo, Brazil. Rev. Inst. Med. Trop. São Paulo.

[21] Garlipp C.R., Bottini P.V., Teixeira A.T. (1995). The relevance of laboratory diagnosis of human cryptosporidiosis and other coccidia. Rev. Inst. Med. Trop. São Paulo.

[22] Franco R.M., Cordeiro N.d.S. (1996). Giardiose e criptosporidiose em creches no município de Campinas, SP. Rev. Soc. Bras. Med. Trop..

[23] Chieffi P.P., Sens Y.A.S., Paschoalotti M.A., Miorin L.A., Silva H.G.C., Jabur P. (1998). Infection by *Cryptosporidium parvum* in renal patients submitted to renal transplant or hemodialysis. Rev. Soc. Bras. Med. Trop..

[24] Cimerman S., Cimerman B., Lewi D.S. (1999). Avaliação da relação entre parasitoses intestinais e fatores de risco para o HIV em pacientes com AIDS. Rev. Soc. Bras. Med. Trop..

[25] Medeiros M.I.C., Neme S.N., Silva P., Capuano D.M., Errera M.C., Fernandes S.A., Valle G.R., Avila F.A. (2001). Etiology of acute diarrhea among children in Ribeirão Preto-SP, Brazil. Rev. Inst. Med. Trop. São Paulo.

[26] Silva C.V., Ferreira M.S., Gonçalves-Pires M.R., Costa-Cruz J.M. (2003). Detection of *Cryptosporidium*-specific coproantigen in human immunodeficiency virus/acquired immunodeficiency syndrome patients by using a commercially available immunoenzymatic assay. Memórias Inst. Oswaldo Cruz.

[27] Cox M.J., Elwin K., Massad E., Azevedo R.S. (2005). Age-specific seroprevalence to an immunodominant *Cryptosporidium* sporozoite antigen in a Brazilian population. Epidemiol. Infect..

[28] Marques F.R., Cardoso L.V., Cavasini C.E., Almeida M.C., Bassi N.A., Almeida M.T.G., Rossit A.R.B., Machado R.L.D. (2005). Performance of an immunoenzymatic assay for *Cryptosporidium* diagnosis of fecal samples. Braz. J. Infect. Dis..

[29] Almeida T.T.C., Pinto P.L.S., Quadros C.M.S., Torres D.M.A.G.V., Kanamura H.Y., Casimiro A.M. (2006). Detection of *Cryptosporidium* sp. in non-diarrheal faeces from children in a day care center in the city of São Paulo, Brazil. Rev. Inst. Med. Trop. São Paulo.

[30] Carvalho T.B., Carvalho L.R., Mascarini L.M. (2006). Occurrence of enteroparasites in day care centers in Botucatu (São Paulo State, Brazil) with emphasis on *Cryptosporidium* sp., *Giardia duodenalis* and *Enterobius vermicularis*. Rev. Inst. Med. Trop. São Paulo.

[31] Mascarini L.M., Donalísio M.R. (2006). Giardiasis and cryptosporidiosis in children institutionalized at daycare centers in the State of São Paulo. Rev. Soc. Bras. Med. Trop..

[32] Costa F.A.C., Gonçalves A.Q., Lassance S.L., Albuquerque C.P., Leite J.P.G., Bóia M.N. (2007). Detection of *Cryptosporidium* spp and other intestinal parasites in children with acute diarrhea and severe dehydration in Rio de Janeiro. Rev. Soc. Bras. Med. Trop..

[33] Rossit A.R.B., Almeida M.T.G., Nogueira C.A.M., Oliveira J.G.C., Barbosa D.M.U., Moscardini A.C., Mascarenhas J.D.P., Gabbay Y.B., Marques F.R., Cardoso L.V. (2007). Bacterial, yeast, parasitic, and viral enteropathogens in HIV-infected children from São Paulo State, Southeastern Brazil. Diagn. Microbiol. Infect. Dis..

[34] Silva M.B.O., Oliveira L.R., Resende J.C.P., Peghini B.C., Ramirez L.E., Lages-Silva E., Correia D. (2007). Seasonal profile and level of CD4+ lymphocytes in the occurrence of cryptosporidiosis and cystoisosporidiosis in HIV/AIDS patients in the Triângulo Mineiro region, Brazil. Rev. Soc. Bras. Med. Trop..

[35] Araújo A.J.U.S., Kanamura H.Y., Almeida M.E., Gomes A.H.S., Pinto T.H.L., Da Silva A.J. (2008). Genotypic identification of *Cryptosporidium* spp. isolated from HIV-infected patients and immunocompetent children of São Paulo, Brazil. Rev. Inst. Med. Trop. São Paulo.

[36] Lucca P.D., De Gaspari E.N., Bozzoli L.M., Funada M.R., Silva S.O., Iuliano W., Soares R.M. (2009). Molecular characterization of *Cryptosporidium* spp. from HIV infected patients from an urban area of Brazil. Rev. Inst. Med. Trop. São Paulo.

[37] Cardoso L.V., Galisteu K.J., Schiesari Júnior A., Abou Chahla L.A.O., Canille R.M.S., Belloto M.V.T., Franco C., Maia I.L., Rossit A.R.B., Machado R.L.D. (2011). Enteric parasites in HIV-1/AIDS-infected patients from a Northwestern São Paulo reference unit in the highly active antiretroviral therapy era. Rev. Soc. Bras. Med. Trop..

[38] Rolando R.F., Silva S.d., Peralta R.H., Silva A.J., Cunha F.S., Bello A.R., Peralta J.M. (2012). Detection and differentiation of *Cryptosporidium* by real-time polymerase chain reaction in stool samples from patients in Rio de Janeiro, Brazil. Memórias Inst. Oswaldo Cruz.

[39] Assis D.C., Resende D.V., Cabrine-Santos M., Correia D., Oliveira-Silva M.B. (2013). Prevalence and genetic characterization of *Cryptosporidium* spp. and Cystoisospora belli in HIV-infected patients. Rev. Inst. Med. Trop. São Paulo.

[40] Gil F.F., Barros M.J., Macedo N.A., Júnior C.G.E., Redoan R., Busatti H., Gomes M.A., Santos J.F.G. (2013). Prevalence of intestinal parasitism and associated symptomatology among hemodialysis patients. Rev. Inst. Med. Trop. São Paulo.

[41] Girotto K.G., Grama D.F., Cunha M.J.R., Faria E.S.M., Limongi J.E., Pinto R.M.C., Cury M.C. (2013). Prevalence and risk factors for intestinal protozoa infection in elderly residents at long term residency institutions in Southeastern Brazil. Rev. Inst. Med. Trop. São Paulo.

[42] Fregonesi B., Suzuki M.N., Machado C.S., Tonani K.A.A., Fernandes A.P.M., Monroe A.A., Cervi M.C., Segura-Muñoz S. (2015). Emergent and re-emergent parasites in HIV-infected children: Immunological and socio-environmental conditions that are involved in the transmission of *Giardia* spp. and *Cryptosporidium* spp.. Rev. Soc. Bras. Med. Trop..

[43] Peralta R.H.S., Velásquez J.N., Cunha F.S., Pantano M.L., Sodré F.C., Silva S., Astudillo O.G., Peralta J.M., Carnevale S. (2016). Genetic diversity of *Cryptosporidium* identified in clinical samples from cities in Brazil and Argentina. Memórias Inst. Oswaldo Cruz.

[44] Adami Y.L., Gama N.A., Cunha F.S., Peralta R.H.S., Lugon J.R. (2025). Presence of *Cryptosporidium* spp. and other enteroparasites with pathogenic potential in hemodialysis patients: An open controlled study. Braz. J. Nephrol..

[45] Newman R.D., Zu S.-X., Wuhib T., Lima A.A.M., Guerrant R.L., Sears C.L. (1994). Household epidemiology of *Cryptosporidium parvum* infection in an urban community in Northeast Brazil. Ann. Intern. Med..

[46] Wuhib T., Silva T.M.J., Newman R.D., Garcia L.S., Pereira M.L.D., Chaves C.S., Wahlquist S.P., Bryan R.T., Guerrant R.L., Sousa A.Q. (1994). Cryptosporidial and microsporidial infections in human immunodeficiency virus-infected patients in Northeastern Brazil. J. Infect. Dis..

[47] Agnew D.G., Lima A.A.M., Newman R.D., Wuhib T., Moore R.D., Guerrant R.L., Sears C.L. (1998). Cryptosporidiosis in Northeastern Brazilian children: Association with increased diarrhea morbidity. J. Infect. Dis..

[48] Newman R.D., Sears C.L., Moore S.R., Nataro J.P., Wuhib T., Agnew D.A., Guerrant R.L., Lima A.A.M. (1999). Longitudinal study of *Cryptosporidium* infection in children in Northeastern Brazil. J. Infect. Dis..

[49] Lima A.A.M., Moore S.R., Barboza M.S., Soares A.M., Schleupner M.A., Newman R.D., Sears C.L., Nataro J.P., Fedorko D.P., Wuhib T. (2000). Persistent diarrhea signals a critical period of increased diarrhea burdens and nutritional shortfalls: A prospective cohort study among children in Northeastern Brazil. J. Infect. Dis..

[50] Brantley R.K., Williams K.R., Silva T.M., Sistrom M., Thielman N.M., Ward H., Lima A.A., Guerrant R.L. (2003). AIDS-associated diarrhea and wasting in Northeast Brazil is associated with subtherapeutic plasma levels of antiretroviral medications and with both bovine and human subtypes of *Cryptosporidium parvum*. Braz. J. Infect. Dis..

[51] Bushen O.Y., Kohli A., Pinkerton R.C., Dupnik K., Newman R.D., Sears C.L., Fayer R., Lima A.A.M., Guerrant R.L. (2007). Heavy cryptosporidial infections in children in northeast Brazil: Comparison of *Cryptosporidium hominis* and *Cryptosporidium parvum*. Trans. R. Soc. Trop. Med. Hyg..

[52] Teixeira M.C.A., Barreto M.L., Melo C., Silva L.R., Moraes L.R.S., Alcântara-Neves N.M. (2007). A serological study of *Cryptosporidium* transmission in a periurban area of a Brazilian Northeastern city. Trop. Med. Int. Health.

[53] Nascimento W.R.C., Cavalcanti I.M.F., Irmão J.I., Rocha F.J.S. (2009). Presença de *Cryptosporidium* spp em crianças com diarreia aguda em uma creche pública de Recife, Estado de Pernambuco. Rev. Soc. Bras. Med. Trop..

[54] Jeske S., Bianchi T.F., Moura M.Q., Baccega B., Pinto N.B., Berne M.E.A., Villela M.M. (2018). Intestinal parasites in cancer patients in the South of Brazil. Braz. J. Biol..

[55] Oshiro E.T., Dorval M.E.C., Nunes V.L.B., Silva M.A.A., Said L.A.M. (2000). Prevalence of *Cryptosporidium parvum* among children of less than 5 years of age in the urban zone of Campo Grande, Mato Grosso do Sul State, Brazil, 1996. Rev. Soc. Bras. Med. Trop..

[56] Pereira M.G.C., Atwill E.R., Barbosa A.P., Silva S.A., García-Zapata M.T.A. (2002). Intra-familial and extra-familial risk factors associated with *Cryptosporidium parvum* infection among children hospitalized for diarrhea in Goiania, Goias, Brazil. Am. J. Trop. Med. Hyg..

[57] Barcelos N.B., Silva L.F.E., Dias R.F.G., Menezes-Filho H.R., Rodrigues R.M. (2018). Opportunistic and non-opportunistic intestinal parasites in HIV/AIDS patients in relation to their clinical and epidemiological status in a specialized medical service in Goiás, Brazil. Rev. Inst. Med. Trop. São Paulo.

[58] Loureiro E.C.B., Linhares A.C., Mata L. (1989). Cryptosporidiosis in children (1–2 years old) with acute diarrhoea from Belém, Pará, Brazil. Memórias Inst. Oswaldo Cruz.

[59] Clemente C.M., Caramori C.A., Padula P., Rodrigues M.A.M. (2000). Gastric cryptosporidiosis as a clue for the diagnosis of the acquired immunodeficiency syndrome. Arq. Gastroenterol..

[60] Souza D.S., Barreiros J.T., Papp K.M., Steindel M., Simões C.M., Barardi C.R. (2004). Comparison between immunomagnetic separation, coupled with immunofluorescence, and the techniques of Faust et al. and of Lutz for the diagnosis of Giardia lamblia cysts in human feces. Rev. Inst. Med. Trop. São Paulo.

[61] Reina F.T.R., Ribeiro C.A., Araújo R.S., Matté M.H., Castanho R.E.P., Tanaka I.I., Viggiani A.M.F.S., Martins L.P.A. (2016). Intestinal and pulmonary infection by *Cryptosporidium parvum* in two patients with HIV/AIDS. Rev. Inst. Med. Trop. São Paulo.

[62] Rezende N.C.C., Bezerra C.L.P.A.M., Almeida J.J.S., Fernandes T.U.G., Luz K.G. (2016). Secondary transmission of cryptosporidiosis associated with well water consumption: Two case studies. Rev. Soc. Bras. Med. Trop..

[63] Barros M.A.F., Navarro I.T., Menezes M.C.N.D., Osaki S.C., Souza C.F. (1994). *Cryptosporidium* sp. em ser humano na zona rural do município de Londrina, Paraná, Brasil: Relato de caso. Semin. Ciênc. Agrár..

[64] Chiuchetta F.A. (2010). Criptosporidiose em paciente com espondilite anquilosante usando adalimumabe. Rev. Bras. Reumatol..

[65] Cabral B.G., Storer J.M., Bono C.S.R., Carrilho C.M.D.M., Pascual J., Tanita M.T., Capobiango J.D., Beraldo E.G., Belei R.A., Neto R.P. (2021). Surto por *Cryptosporidium* spp em unidade de terapia intensiva: Medidas de controle. Braz. J. Infect. Dis..

[66] Rosendo R.Z., Bianchini A.J., Corrêa de Barros R., Breda G.L., Lotha G. (2023). Disseminated cryptosporidiosis in a child submitted to hematopoietic stem cell transplantation for CD40 ligand deficiency: Case report. Braz. J. Infect. Dis..

[67] Jann H.W., Cabral-Castro M.J., Costa J.V.B., Alencar A.C.M.B., Peralta J.M., Peralta R.H.S. (2022). Prevalence of human cryptosporidiosis in the Americas: Systematic review and meta-analysis. Rev. Inst. Med. Trop. São Paulo.

[68] Sidone O.J.G., Haddad E.A., Mena-Chalco J.P. (2016). Science in Brazilian regions: Evolution of production and collaboration networks. Transinformação.

[69] Afzal M., Agarwal S., Elshaikh R.H., Babker A.M.A., Osman E.A.I., Choudhary R.K., Jaiswal S., Zahir F., Prabhakar P.K., Abbas A.M. (2025). Innovative diagnostic approaches and challenges in the management of HIV: Bridging basic science and clinical practice. Life.

[70] Smith R.P., Clifton-Hadley F.A., Cheney T., Giles M. (2014). Prevalence and molecular typing of *Cryptosporidium* in dairy cattle in England and Wales and examination of potential on-farm transmission routes. Vet. Parasitol..

[71] Morris A., Robinson G., Swain M.T., Chalmers R.M. (2019). Direct sequencing of *Cryptosporidium* in stool samples for public health. Front. Public Health.

[72] Costa D., Soulieux L., Razakandrainibe R., Basmaciyan L., Gargala G., Valot S., Dalle F., Favennec L. (2021). Comparative Performance of eight PCR methods to detect *Cryptosporidium* species. Pathogens.

[73] Dabas A., Shah D., Bhatnagar S., Lodha R. (2017). Epidemiology of *Cryptosporidium* in Pediatric Diarrheal Illnesses. Indian Pediatr..

[74] Colford J.M., Tager I.B., Hirozawa A.M., Lemp G.F., Aragon T., Petersen C. (1996). Cryptosporidiosis among patients infected with human immunodeficiency virus: Factors related to symptomatic infection and survival. Am. J. Epidemiol..

